# The leakage effect may undermine the circular economy efforts

**DOI:** 10.1038/s41598-023-44004-x

**Published:** 2023-10-04

**Authors:** Karolina Safarzynska, Lorenzo Di Domenico, Marco Raberto

**Affiliations:** 1 Faculty of Economic Sciences, University of Warsaw, Dluga 44/50, 00-241 Warsaw, Poland; 2 Catholic University of the Sacred Heart, Via Necchi 5, 20125 Milan, Italy; 3 University of Genoa, Via Opera Pia 15, 16145 Genoa, Italy

**Keywords:** Environmental impact, Socioeconomic scenarios, Sustainability

## Abstract

In this paper, we examine the impact of the circular economy on global resource extraction. To this end, we make an input–output analysis dynamic by combining it with an agent-based model of the capital sector. This approach allows us to study the evolution of the circular economy due to the endogenous decisions of firms on whether to invest in the capital expansion of primary or secondary sectors. Previous studies have examined the macroeconomic effects of the circular economy using scenarios that exogenously impose higher recycling rates, improved resource efficiency, or lowered demand on the economy. Such studies typically assume static consumer budgets, no price adjustments, capital investments in recycling infrastructure, or technological innovation. We relax these assumptions in a novel agent-based input–output model (ABM-IO). We show that the circular economy can significantly reduce the extraction of iron, aluminum, and nonferrous metals if implemented globally. However, the leakage effect may also cause some metal-intensive industries to relocate outside the EU, offsetting the circular economy efforts. The risk of the leakage effect is especially high for copper.

## Introduction

The urgency to reduce the depletion of natural resources is indisputable. An eightfold increase in global resource extraction and a doubling of per capita material consumption have been observed over the last century^1^. In the future, the impact of the low-carbon transition on resource extraction is expected to be immense and exacerbate this trend^2^. The demand for rare metals and minerals needed for technologies such as solar photovoltaics, batteries, electric vehicles, wind turbines, fuels cells, and nuclear reactors is expected to increase between 1000 and 87,000%, depending on the specific technology, by 2060^3^. Today’s mineral supply and investment plans are insufficient for the transformation of the energy sector^4^. Many minerals critical for emerging technologies are in scarcity due to political tensions, shortages, and declining grades of metal ores^5, 6^. Another difficulty is that many critical metals are obtained only as by-products of the mining of other metals. For instance, nearly all indium production occurs as a by-product of zinc^7^. As a result, supply of such metals is inelastic and unable to respond quickly to supply shortages.

The transition to a circular economy (CE) has been proposed as a possible solution to these problems that can reduce waste, carbon dioxide emissions, and global resource extraction^8, 9^. It has been high on the political agenda. For instance, the EU introduced the Circular Economy Action Plan that set targets for landfill, reuse, and recycling to be achieved by 2035. The new monitoring framework was adopted to track progress in improving material and consumption footprints, resource productivity, and reducing greenhouse gas emissions and material dependency^9^. So far, there is no single definition of the circular economy, which is often perceived as a combination of reduce, reuse, and recycle^10^. Other definitions emphasize the need for the more efficient use of products and processes, extending product lifespan, finding new applications for used materials/old products, as well as changing business models, and how production and consumption are organized^11, 12^.

Most models of the circular economy focus on its two aspects: increasing recycling rates and improving resource efficiency^12–14^, which typically have been studied using static input–output analysis (other studies use Life-Cycle Assessment (LCA), for an overview see Towa et al.^15^). Studying the CE using the static input–output analysis offers an important first step in understanding its economy-wide impacts. However, the approach suffers from three problems when it comes to making projections about future resource extraction. First, in most studies, secondary and primary products are perfect substitutes, characterized by the same price. Second, secondary inputs substitute for primary production in an exogenously-specified scenario that defines the share of recycled materials in production. No capital investment is considered, although the lack of related infrastructure has been a major barrier to the expansion of secondary production in many sectors. Without capital investment in new infrastructure, it is not possible to increase recycling rates or implement new business models to support the transition to the circular economy^16, 17^. Finally, the static input–output analysis ignores macroeconomic dynamics, e.g., endogenous demand or capital formation, monetary and fiscal policy.

Because of these limitations, static input–output models are ill-equipped to study the leakage and rebound effects as a result of the circular economy efforts. However, there are concerns that the proliferation of cheap products made from recycled materials could boost demand, offsetting reductions in energy use and the extraction of raw materials, referred to as the circular economy rebound effect^18, 19^. The leakage effect refers to the relocation of industries from developed to developing countries as a result of stringent environmental policies that increase prices in developed countries^20^. This effect has achieved little attention so far in the context of the circular economy. Yet, it has been shown that the waste embodied in trade increases faster than waste generated domestically^21^. Giljum et al.^22^ find that between 1997 and 2007, global material extraction directly or indirectly related to the production of traded products increased by 34%. Europe ranked second as the most important destination for such imported materials.

In this paper, we relax the above-discussed shortcomings of static input–output analysis in a novel model of the circular economy to examine the leakage and the rebound effect. We consider two regions: the EU and non-EU (NEU). As a novelty of our approach, we make input–output analysis dynamic by linking it to an agent-based model of the capital sector. We use the hybrid EXIOBASE database, which is a global Multi-Regional Input–Output Table (MR-IOT), to study the physical flows of resources within and between regions^23–25^. The EXIOBASE database dominates in input–output studies of the circular economy. It was developed by a consortium of several European research institutes by harmonizing and detailing supply-use tables for a large number of countries. The database is unique in that it provides detailed accounts of inputs used for the primary and secondary production of wood, pulp, paper, plastic, glass, steel, precious metals, aluminum, ‘lead, zinc and tin’, copper, other nonferrous metals, and construction. It exists in two forms. In the monetary form, transactions in the supply-use tables are recorded in monetary units, while in the hybrid form, they are specified in mass or energy units. Most input–output studies of the circular economy (75%) use the monetary version of the EXIOBASE for the latest year available and project it into the future^26–28^. Exceptions include: a model of the Belgium economy^26^, or a study of plastic policies in the EU^27^, which use hybrid input–output tables (IOTs). Here, we follow the latter approach. The advantage of the hybrid IOTs is that policy goals for the circular economy can be studied directly, as these are typically expressed in physical units^15, 28^.

In our model, each sector in the input–output table (IOT) is modelled as a representative firm, whose output is determined using the Leontief inverse matrix, as in classical input–output analysis. Contrary to static input–output analysis, each firm invests in the capital stock that determines its maximum production. To expand production, firms order capital goods in the capital market. Capital constraints act as supply shocks and may propagate through production networks. In the capital market, heterogeneous capital firms engage in R&D activities to improve the productivity of the capital (machinery) they offer to firms in different sectors. In this setting, the circular economy evolves because of endogenous decisions of firms, who decide whether to invest in new capital to produce output from virgin or recycled material in order to minimize their total production costs. In particular, firms offer homogenous products sold at one price, but which can be produced using two techniques, i.e., from primary or secondary inputs.

Endogenizing capital investment in an input–output analysis has two effects on the economy. First, the larger the share of secondary production in total output, the lower its price. This follows from the assumption that firms only invest in expanding secondary production if this lowers their unit costs of total production. Second, the proliferation of products made from recycled materials affects prices of all products in the economy, and this way total supply and demand. In existing input–output studies of the CE, recycling rates do not affect prices. Relaxing this assumption allowed us to examine the leakage effect in the circular economy. Tan et al.^29^ identified three channels behind the (carbon) leakage effect in the Computable General Equilibrium (CGE) model: (1) the competitiveness channel, (2) the demand channel, and (3) the energy channel. The competitiveness channel captures that stringent climate policy in one region may weaken the competitiveness of its carbon-intensive sector by increasing imports or decreasing exports. The demand channel indirectly affects production and emissions by altering income levels, and product prices, and thus domestic and foreign demand. Finally, the energy channel results from reduced fossil fuel use in emission-abating regions, which depresses energy prices, and increases consumption and emissions in other regions. In our model, we consider the ‘material’ channel instead of the energy channel because we focus on policies that reduce resource extraction. Similar to the energy channel, the material channel affects the leakage effect by affecting material prices in the region that implemented the CE.

In all model simulations, the demand and ‘material' channels are always present. However, our results show that these channels are insufficient to cause the leakage effect in scenarios, where consumers spend constant shares of their budgets on goods from different sectors and regions, as in the static input–output analysis. We also consider the ‘evolving budget’ scenario, where consumers can choose from which region to buy products depending on their relative prices. This scenario results in an additional ‘leakage’ channel, i.e., the competitiveness channel. In particular, in the ‘evolving budget’ scenario, budget shares are constant across sectors, but goods from the same industry are substitutable across regions. We show that relaxing the assumption of constant budget shares causes the relocation of some metal-intensive industries outside the EU, increasing the global extraction of metal ores, e.g., copper. This finding is consistent with the results of Tan et al.^29^, who show that the competitiveness channel is the main source of the leakage effect.

We use an agent-based modeling (ABM) technique that has been increasingly popular in economic studies^30, 31^. The method allows for the relaxation of assumptions commonly used in economic models for the purpose of analytical tractability. In ABMs, macro patterns emerge from the interactions of heterogeneous agents. Such interactions are characterized by increasing returns, learning, and path dependence. Agents are described by behavioral rules that may evolve over time. ABMs have enriched our understanding of clustered volatility or systemic risk in the financial markets^32, 33^; business cycles and industry dynamics^34–37^; or climate change impacts^38, 39^. Initially, economic agent-based models have been used to study the mechanisms behind macroeconomic phenomena, but not for forecasting. This relates to the fact that such models have a large degree of freedom and require many parameters to be calibrated. The recent progress in computing power and data collection has led to the development of ABMs of national economies populated by a large number of interacting agents, whose characteristics are parametrized using micro and macro data from national accounts, sectoral accounts, input–output tables (IOT), government statistics, and census and business demography data.^35, 40, 41^ Recently, an agent-based model of the Austrian economy by Poledna et al.^40^ has been shown to outcompete the Vector Autoregression (VAR) and Dynamic Stochastic General Equilibrium (DSGE) models in out-of-sample forecasting of macro variables. Following this line of research, we combine input–output analysis with agent-based modelling of the capital market. Our approach offers the advantage of our results being comparable to existing models of the CE using the EXIOBASE, while it brings macroeconomics to the analysis, namely: capital investment, endogenous demand, trade, and price adjustments. We use it to study the global extraction of virgin materials as a result of the circular economy.

## Results

**Figure 1:**
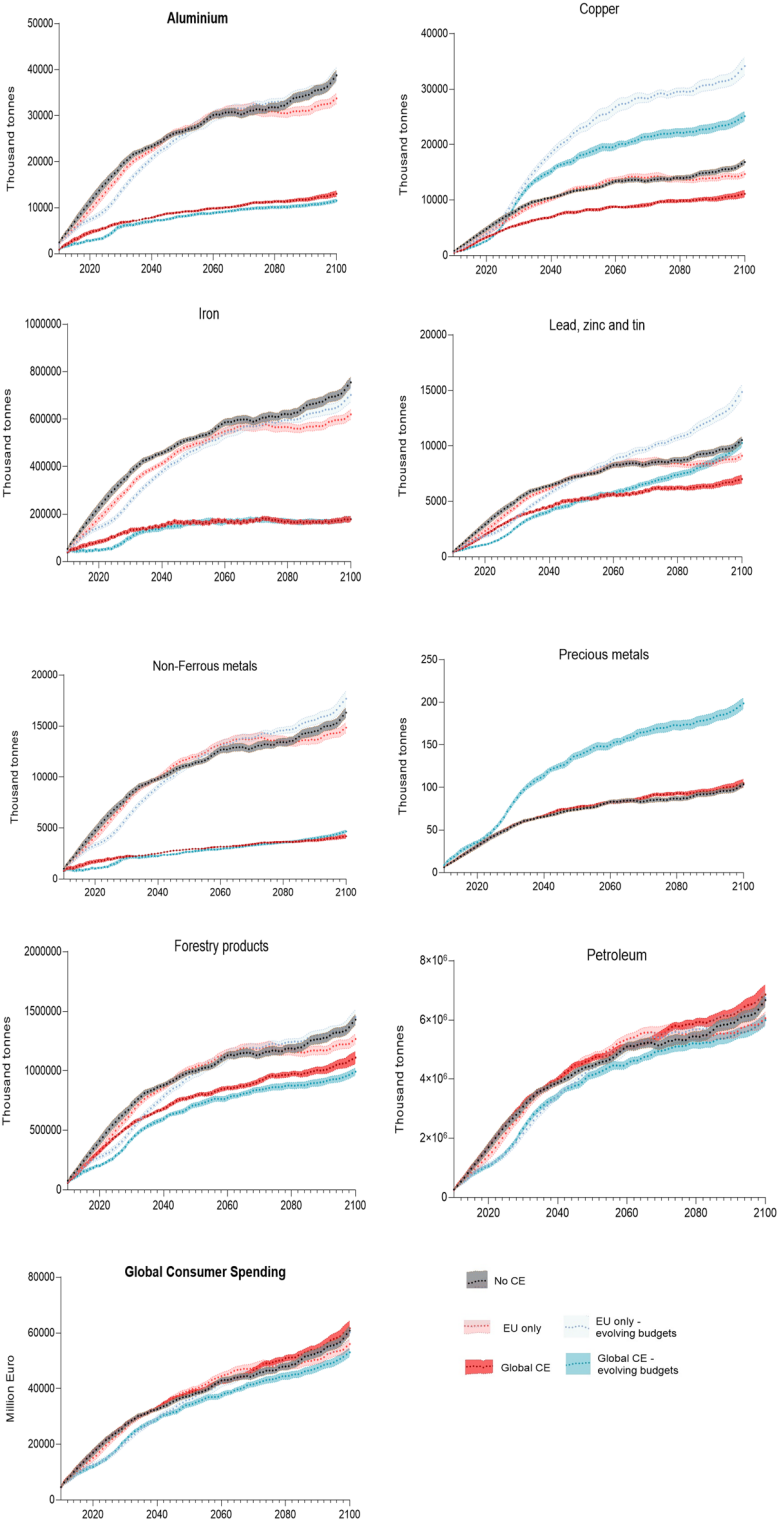
The patterns of global primary production. The relative reduction in global primary production is significant for aluminum, iron, and nonferrous metals if the CE is implemented globally (dark red or dark blue lines) compared to the baseline (no-CE) scenario (black lines). The relative reduction is moderate for forestry products. The primary production of copper, ‘lead, zinc, and tin', and precious metals increases relative to the baseline under the evolving budget scenario (blue lines). Each line presents the mean values from 100 simulations that come from Monte Carlo analysis, i.e., model simulations run with the same parameter values but different seeds for generating random numbers. Shaded areas represent the error bars. They are calculated as standard errors, i.e., the standard deviation of these means multiplied by 1/√n, where n is the number of observations, which here is 100. The figures present the moving averages over 10 years to smooth the lines. The global production of primary sectors is measured in thousand tons and global consumer spending in millions of Euros. We refer to the ‘no CE’ scenario as the baseline in the text.

In model simulations, each period corresponds to a single year. We study model dynamics between 2010 and 2100. In Supplementary Table 4 (ST4), we report the mean values of selected variables from 100 model simulations, conducted for the same parameter setting so as to control for the presence of stochastic factors in our model. We compute the mean value of each variable for 2050–2100, during which period the model dynamics have stabilized. Figure 1 illustrates the dynamics of the main variables over time.

In our simulations, in the absence of recycling, the patterns of natural resource extraction depend on two factors: the technical coefficients of the IOT and the dynamics of total demand. In the previous studies, the vector of total demand was usually adjusted exogenously to match the desired scenario. Contrary to this, in our approach, demand is endogenous and depends on economic growth that is driven by improvements in capital productivities. We calibrated the annual growth of the capital productivity frontier so that the rate of growth of consumption per capita between 2010 and 2100 was equal on average to 1.6% for a depreciation rate of δ = 10%, which is consistent with the SSP1 growth scenario^42^ (see also the Calibration Section of the Supplementary Materials). We are interested in the relative reduction in primary production between scenarios with and without recycling.

In the baseline scenario, which we also refer to as the ‘no CE’ scenario, we do not consider capital investment in the secondary sector. We use this scenario as a baseline to which we compare the relative reduction in resource extraction due to the CE in different scenarios. We study the impact of recycling in scenarios that differ with respect to: (1) whether the CE is implemented only in the EU or globally in the ‘CE only EU’ and ‘global CE’ scenarios, respectively; and (2) whether the shares of consumer budgets are constant or change depending on relative prices between regions. In the baseline scenario, shares are constant, while goods produced in different regions, but in the same sector, are not substitutable. We consider two additional scenarios with ‘evolving budget shares’, in which consumers shift their expenditures between goods produced in two regions depending on their relative prices. We keep the total share of consumers’ budget spent on goods from a specific industry constant, but consumers buy increasingly more goods over time from the region where they are cheaper (see Fig. 4). In particular, we consider ‘slow updating’ and ‘fast updating’ of consumer budget shares, which differ with respect to the speed of the shifting of consumer expenditures between regions. In these scenarios, we assume that there are contingencies that limit the possibilities for increasing production in the short term, e.g., firms need to expand their production capacity to respond to the fluctuation in demand, or myopia and habits on the side of consumers slow down the adjustments in consumption patterns in response to price changes. Formally, we use a replicator-dynamic equation to model changes in the composition of demand^43^. In the ‘slow updating’ scenario, the parameter describing the speed of demand adjustments to price changes is lower than in the ‘fast updating’ scenario. These two scenarios differ only in how quickly budget shares respond to price changes, which affects the magnitude of export/import changes.

To check the robustness of our results, we report the results from an additional ‘Global CE—50%’ scenario, where we impose a rule that firms invest in the capital expansion of the secondary sector to ensure that 50% of their output is produced from recycled materials. In this scenario, we assume no updating of consumer budget shares. This scenario comes the closest to previous studies of the CE.

Table 1 summarizes the ratios of mean production in selected industries from 2050 to 2100 in the ‘no CE’ scenario with no recycling relative to their mean production in different scenarios. We report the ratios for selected metal sectors (primary production), fossil fuel, forestry products, electricity, and global consumer spending. A ratio greater than 1 means a relative decrease in primary production due to the CE compared to the baseline scenario. A ratio less than 1 indicates the opposite, i.e., a relative increase in resource extraction. The table shows that the results from the ‘Global CE -50%’ scenario are close to the findings from Wiebe et al.^23^. The authors report that if 65% of global output is produced using secondary materials, metal extraction will be reduced by 10–27% by 2030 compared to the baseline. Similarly, in our model simulations, the relative reduction in metal extraction in the ‘Global CE—50%’ scenario by 2100 compared to the baseline scenario without recycling is between 8 and 30% (Table 1).

**Table 1:**
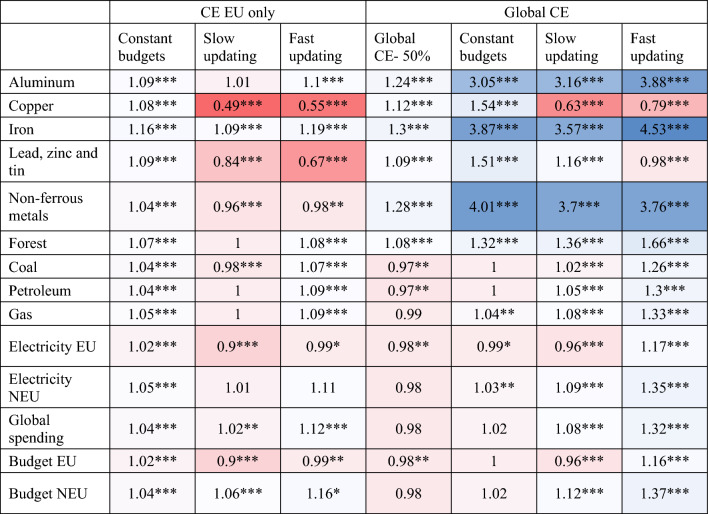
Total primary production (tons) in the non-CE economy relative to total primary production (tons) in the CE, depending on the speed of updating of consumer budget shares and which region implements the CE (EU vs global).

The ratio > 1 indicates a relative reduction in the primary production due to the CE. The shading of the cells in the table indicates the magnitude of the effect. The greater the decrease in resource extraction compared to the baseline scenario (‘no CE’), the darker the blue color, while the greater the increase in extraction, the darker the red color.

***Indicates if there are statistically significant differences between each scenario and the baseline at the 0.1% level; ** at 5% and * at 10% level, using the Mann–Whitney test. Data behind the analysis can be found in ST 1.

The results of model simulations with constant budget shares can shed light on the circular rebound effect. If budget shares are constant, a ratio of less than one indicates an increase in resource extraction in the circular economy due to higher final demand compared to the baseline scenario (the demand and material channel). Under evolving budget shares, a ratio of less than one may be caused by changes in total demand or the relocation of industries (the competitiveness channel). It is important to emphasize that in our model, the competitiveness channel is caused only by changes in the composition of the final demand by consumers and not by changes in the shares of intermediate inputs. In other studies of the leakage effect, firms adjust the share of inputs purchased from different regions when climate policy affects input prices. This is not the case in our model. Our assumption is motivated by the fact that changing intermediate inputs in production due to environmental policy would affect the coefficients of the input–output table and make our results incomparable to the previous studies on the circular economy using this method.

The results in Table 1 show that, under constant budget shares, the rebound effect is unlikely if the EU is the only region implementing the CE. This is supported by all reported ratios being greater than 1 (2nd Column). We find that the relative primary production of most resources is reduced by 4% to 16% compared to the baseline scenario (2nd Column). If the CE is implemented globally under constant budget shares, the global circular economy reduces the extraction of aluminum, iron, and nonferrous metals by as much as three times relative to the baseline. All in all, our results do not support the circular rebound effect in material or energy use (6th Column). The only exception is the 'Global CE—50%' scenario, in which the circular economy causes an energy rebound, i.e., a relative increase in gas, coal, and electricity use compared to the baseline scenario.

The primary production of aluminum, iron, and nonferrous metals will decline more than threefold by 2100 if the circular economy is implemented globally compared to the baseline scenario, regardless of consumer behavior (Columns 6–8). In these sectors, the scarcity of scrap prevents the achievement of 100% production from recycled materials. The relative reduction in the production of forestry products, copper, or ‘lead, zinc and tin’, compared to the baseline scenario, lies between 32 and 54% in simulations with constant budget shares (6th Column). However, our results also indicate that these metals may be prone to the leakage effect. In particular, in the ‘evolving budget’ scenarios, where consumers choose between commodities produced in the EU and NEU, the primary production of these metals increases compared to the baseline scenario with no recycling. The risk of the leakage effect is greater if only the EU undergoes the circular transition. In particular, a relocation of metal-intensive industries outside the EU, where production is more copper and ‘lead, zinc and tin’-intensive, increases the primary production, and thus extraction, of these metals (4-5th Columns). If the CE is introduced globally, recycling in the NEU attenuates this effect as a larger share of global production becomes supplied by the secondary sector (Columns 7–8).

### Closing the wage gap between regions increases resource extraction

**Figure 2:**
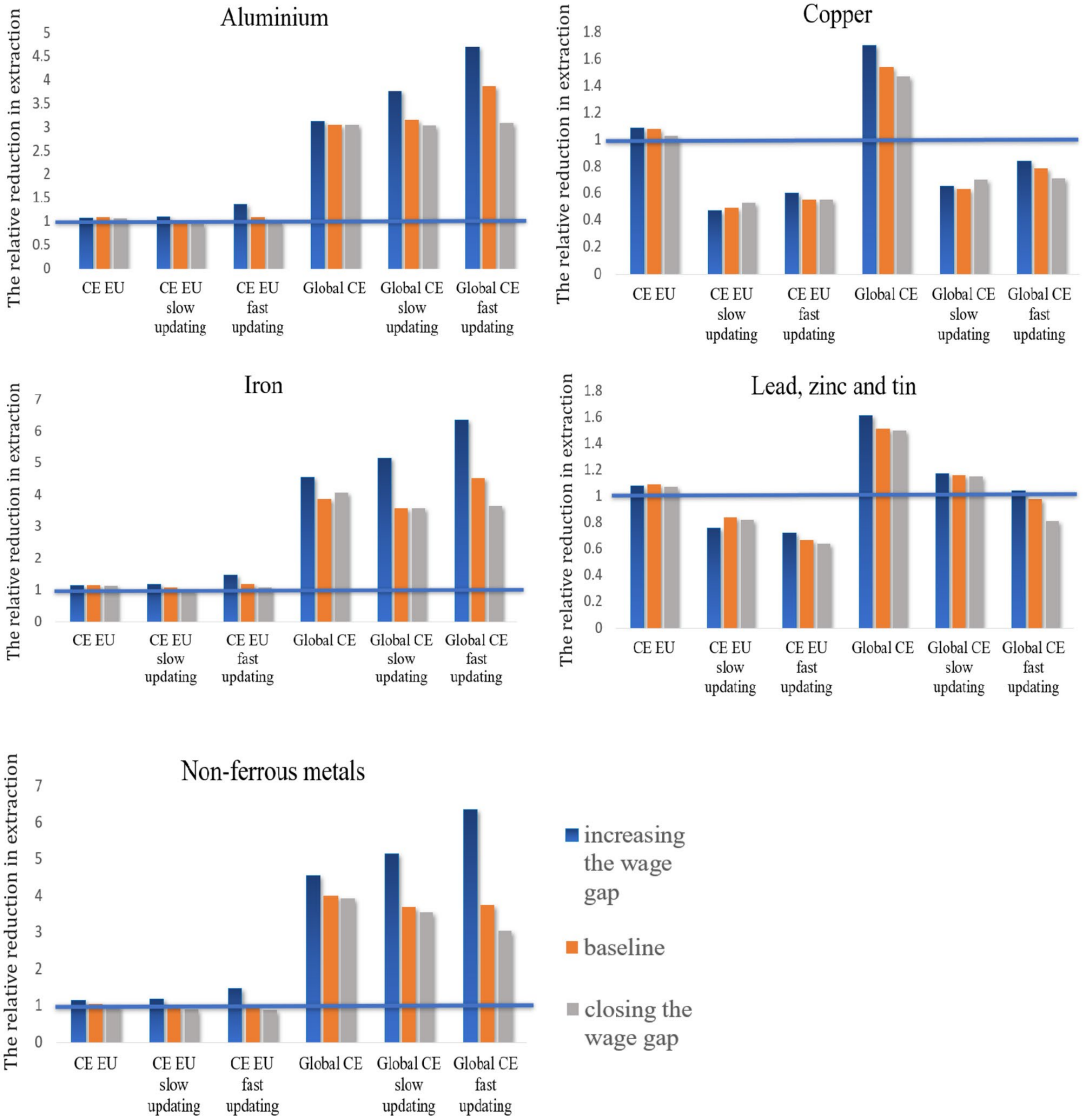
The relative reduction in the primary production of metals in scenarios characterized by different wage dynamics between regions, compared to their primary production in the baseline scenario with no-CE, where wages grow at the same rate. A horizontal blue line indicates a ratio, below which the CE causes an increase in raw material extraction, and above which it reduces resource use. For almost all metals, the relative reduction in the primary production of metals is greatest when the wage gap is present (blue bars). Closing the wage gap (grey line) results in a smaller relative reduction in primary production compared to the baseline scenario. The leakage effect occurs for copper under the ‘evolving budget’ scenario in the event that the CE was implemented in the EU or globally; for ‘lead, zinc and tin’, the leakage effect occurs in the ‘evolving budget’ scenario when the CE is implemented only in the EU but not globally.

In the scenarios with ‘evolving budget shares’, relative wages in the EU and NEU influence consumers’ decisions about which products (from which region) to buy, and thus the materials embodied in international trade. So far, the large body of empirical research on regional convergence has not formulated a decisive answer to the question of whether wage convergence across regions is feasible^44^. Therefore, we compare the results from model simulations with and without convergence. In the baseline scenario, the parameter describing the response of wages to changes in the employment rate was set to be the same in both regions. We conduct additional model simulations, where we relax this assumption: (1) the ‘increasing the wage gap’ scenario, in which this parameter is greater in the EU compared to the NEU (see Supplementary Tables 5a&b); and (2) the ‘closing the wage gap’ scenario, where it is greater in the NEU, making NEU wages grow faster compared to the EU (Supplementary Tables 6a&b). Figure 2 compares the ratios of primary production in the baseline scenario with no CE to the scenario where the CE was introduced either globally or in the EU only, depending on the dynamics of relative wages between regions. It is important to note that the baseline scenario with no CE, to which we compare the results in this section, differs from the one in Table 1. In particular, in Fig. 2 we compare the results from each scenario to model simulations with no recycling, but additionally characterized by the same wage dynamics as the scenario under study.

In Fig. 2, the blue bars indicate the relative primary production in model simulations with the wage gap increasing between regions compared to the scenario with no-CE, where wages grow at the same rate. The grey bars do the same for the scenario where the wage gap between regions is closing, while the orange bars represent the scenario where wages grow at the same rate. If the bar is below 1, it indicates the leakage effect, i.e., that the primary production is relatively greater in a given scenario compared to the baseline. The figure shows that for almost all metals, the relative reduction in primary production of metals is largest when the wage gap is present. Closing the wage gap results in a smaller relative reduction in primary production compared to the baseline scenario.

In particular, when wages increase relatively more in the EU compared to the NEU in the 'increasing the wage gap' scenario, this increases the consumption of foreign goods in the EU while decreasing the consumption of domestic goods. In other words, rising wages in the EU relative to the NEU lead to a shift in real consumer spending from the EU to the NEU over time (even under fixed budget shares). The circular economy reduces resource extraction in the ‘increasing the wage gap’ scenario more, compared to the baseline scenario with wages growing at the same rate in both regions, as the material intensity of production is higher on average in the EU than in the NEU for most metals. As a result, the higher the recycling rates in the NEU, the lower the prices therein, reinforcing the relocation of real consumer expenditures from the EU to NEU, which reduces resource extraction.

There are two exceptions. In particular, production outside the EU is more copper and ‘lead, zinc, and tin’-intensive than in the EU. If consumer budget shares are constant, the primary production of copper decreases slightly if the CE is implemented only in the EU and decreases significantly if the CE is implemented globally. In case the EU also adopts the CE, the prices of materials, and thus final products, decrease, limiting the reallocation of demand from EU to non-EU countries, where production is more copper-intensive. However, under the evolving consumer budgets, the leakage effect occurs, regardless of whether the CE is implemented in the EU or globally. For copper, the slow and fast updating does not affect the results, i.e., the speed of the adjustments in consumer demand has an insignificant impact on the size of the leakage effect. For ‘lead, zinc, and tin’, increasing the speed of adjustments in consumer demand changes the results from a reduction in the extraction of these metal extraction to an increase. In general, the reallocation of demand from the EU to non-EU countries increases the demand for ‘lead, zinc, and tin’, as the production outside the EU is relatively more intensive for these metals than in the EU. If reallocation is slower, the demand for these metals is satisfied using recycled materials. For fast updating, the demand for ‘lead, zinc and tin’ exceeds the supply of recycled metals, which explains an increase in the primary production of these metals compared to the baseline*. *The effect depends on the coefficients of the input–output table. In reality, the speed of adjustments in demand in response to price changes depends on many factors, i.e., consumer habits, how quickly firms respond to demand fluctuations by expanding their production capacity, and on trade barriers. In this context, slow updating may present a more realistic scenario.

## Discussion

Under EU regulations, the EU countries have committed to reducing greenhouse gas emissions by 55% by 2030, which would make the EU the first continent to achieve climate neutrality by 2050. Increasing recycling rates to 65% is one of the pillars of the EU's climate policy. The input–output analysis is a popular method for assessing the economy-wide and environmental impacts of the CE. Using this method, Wiebe et al.^27^, for example, find that the implementation of the CE globally can help reduce metal extraction by 27%, fossil fuel extraction by 8%, the production of forestry products by 8%, and of non-metallic minerals by 7%, while increasing employment due to sectoral adjustments, compared to the baseline scenario with no recycling.

Typically, studies using an input–output analysis assess changes in resource extraction over the next few years, but not over longer time horizons. The reason for this is that such an approach lacks macroeconomic dynamics. Therefore, its main purpose is to study sectoral adjustments as a result of exogenously specified changes, for instance, to the vector of final demand. We introduce macroeconomics into this analysis by linking input–output tables to an agent-based model of the capital sector. This allows us to model investment and final demand, capital formation and innovation as endogenous to the model dynamics, which drive economic growth. We calibrated the parameters of the agent-based model so that economic growth is in line with the SSP1 growth projection^45^. Our model simulations generate similar results to the previous estimates in the scenario, where we impose a rule that firms produce 50% of their output from recycled materials and assume static budget shares. The results of the model simulations in which we endogenize capital investment predict greater reductions in resource extraction. In particular, the global circular economy can reduce the extraction of aluminum, iron, and nonferrous metals by a factor of three by 2100. The introduction of the circular economy in the EU alone can lead to a 2–10% reduction in global material extraction by that year compared to the baseline model simulations without recycling.

The results of our model simulations with ‘evolving budget shares’ show that the leakage may undermine the circular economy efforts in some industries. In particular, we find that a shift of production outside the EU can increase the extraction of copper, and ‘lead, zinc and tin’, offsetting the reduction in their extraction due to higher recycling rates. Studying this effect is important as waste and materials embodied in trade have increased dramatically in recent decades^24, 25^. We find that copper extraction is 21% higher in the global CE economy, under ‘evolving budget shares’, compared to the baseline scenario with ‘no CE’ between 2050 and 2100 (8th Column in Table 1). This may threaten the future of low-carbon technologies. Copper is used in more than eight clean energy generation and storage technologies, including solar PV and wind power, as well as in the transmission infrastructure needed to connect these new technologies to electricity grids^45^. We are not the first to predict a large increase in the extraction of copper. For example, Elshkaki et al.^46^ have shown that copper demand is expected to increase by 275% to 350% by 2050, exceeding reserves by 2040. The authors use regression analysis to predict the impact of economic growth between 1980 and 2010 on copper demand. Using stock dynamics and regression approaches, Schipper et al.^47^ estimate that demand for copper will increase by a factor of 3–21 between 2015 and 2100. All in all, our findings provide additional justification for implementing the circular economy in most industries, while showing that for few selected industries caution is needed as the circular economy only brings a reduction in resource extraction if there are limits to trade.

In future research, it is important to examine the rebound and leakage effects in the input–output model of the CE that goes beyond two regions, incorporates macroeconomic dynamics, and endogenous changes to technical coefficients. Making a household sector composed of heterogeneous consumers would allow for a more in-depth analysis of future patterns of resource extraction in response to changes in consumption, e.g., due to lifestyle changes, and by different socio-economic groups. Consumer heterogeneity can be introduced into input–output analysis by linking IOTs to the microsimulation of household expenditures (using data from household budget surveys) or an agent-based model of the household sector. Finally, including multiple countries in input–output agent-based models would allow for the analysis of exchange rates, the balance of payments, and trade costs in the future. These factors could not have been meaningfully studied with our approach because of the aggregation of data at the level of two regions.

## Methods

In our model, we consider two regions: the European Union (EU) and outside the EU, to which we refer as non-EU (NEU). In both regions, representative consumers receive wages and pay taxes. Consumers allocate their disposable income to goods from different sectors. Aggregate demand is the sum of consumer demand, investments, exports, and government orders in both regions. In particular, governments in the EU and NEU collect taxes and order products from each industry. Once aggregate demand has been determined, the output of each industry is calculated using the Leontief inverse matrix.

**Figure 3:**
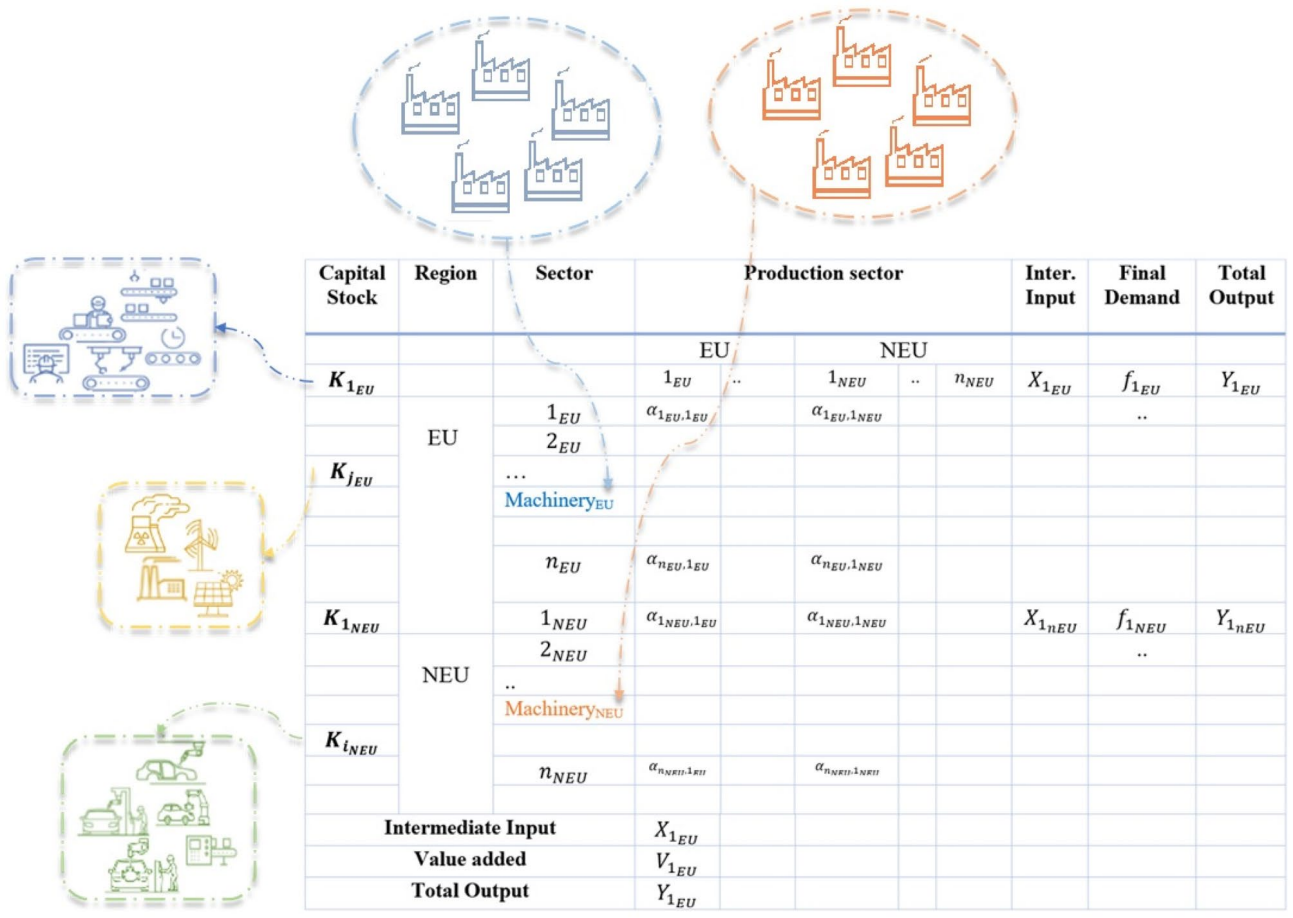
The structure of the ABM-IO model: the capital sector is modelled as an agent-based model that corresponds to the ‘Machinery and equipment’ sector in the EXIOBASE database; firms in each sector face capital constraints, if firms want to expand their production, they need to order capital goods on the capital market; capital goods then arrive in the next period increasing the sector-firms’ maximum production. Capital stock in each sector is composed of different capital vintages. In sectors (e.g., aluminum), where firms can choose whether to produce goods using the primary or secondary technique (e.g., aluminum ore versus re-processed aluminum), they invest in the capital expansion of these sectors accordingly.

The model was run using Laboratory for Simulation Development (LSD) software. The codes can be found at https://osf.io/3vdpr/. Figure 3 shows the structure of our model. In contrast to classical input–output analysis, we introduce constraints on the maximum output ${(Q}_{s}^{max})$ that can be produced every year in each sector *s*, which is determined by the firms’ capital stock. In particular, each sector-firm of the input–output table (IOT) adjusts productive capacity in order to satisfy expected demand by investing in capital goods. Capital goods can be thought of as machinery. Each firm owns a stock of vintage capital goods purchased at different times. Capital goods depreciate at a constant rate. If a firm cannot produce the desired output due to insufficient capital, it will not be able to satisfy its total demand. In this case, its available output is distributed among all sectors of the economy according to the rationing algorithm (described below), while the firm orders capital goods on the capital market. The capital is delivered in the next period.

The capital market is modelled using an agent-based model that corresponds to the 'Machinery and equipment' sector in the IOT. The design of the capital market is based on a seminal work by Nelson and Winter^48^ and Dosi et al.^49^. Our contribution is to link IOTs with this kind of model. The capital market is composed of many heterogeneous capital firms that differ in terms of: (1) own labor productivity, which describes how many low, medium, and high-skilled workers they require to produce one unit of the capital good; and (2) the productivity of the capital good (*A*) offered to firms-sectors of the IOT. Capital firms constantly engage in R&D activities to improve the productivity of their own technology as well as the productivity of capital goods offered to their clients. The success of the R&D activities is uncertain as these activities are modelled as stochastic processes. The emergent property of firms’ interactions in the capital market is technological progress. As a result, firms in different sectors order more productive capital over time, characterized by higher capital productivity. Finally, in our approach, which distinguishes our work from previous agent-based models, capital firms use other inputs than labor to produce capital goods, according to the technical coefficients of the IOT corresponding to the ‘Manufacture of machinery and equipment’ sector.

Another novelty of our approach concerns capital investment in the primary and secondary sectors. EXIOBASE distinguishes the following sectors that can produce output using virgin or recycled materials: wood, pulp, paper, plastic, glass, steel, precious metals, aluminum, ‘lead, zinc and tin’, copper, other non-ferrous metals, and construction. Firms-sectors in the IOTs, which can produce output using virgin or raw materials, decide whether to invest in the capital expansion of the primary or secondary sectors based on their unit costs. They order capital goods accordingly. These decisions determine both recycling rates and prices in the economy. After the output of each industry has been determined, employment in each industry and entrepreneur profits are computed. Both are distributed as income to consumers in each region and constitute consumer spending. The latter is allocated across different sectors as final demand according to the vector of household budget shares. Afterward, the afore-mentioned sequence of events is repeated.

### Consumer decisions

In each period *t*, the total consumer expenditures/budgets ${b}_{kt}$ in region *k* are allocated across commodities produced in different industries and regions, according to the vector of household budget shares ${G}_{k}$. In particular, its entries ${g}_{k,{s}_{j}}$ define the share of total budget that consumers in region *k* spend on goods from sector *s* produced in region *j* (${s}_{j}$). We assume that the vector of budget shares differs between regions but is the same for all consumers within a region. In particular, the vector of household budget shares in the EU (*G*_*EU*_) and outside the EU (*G*_*NEU*_) are calibrated using the monetary EXIOBASE from 2011.

In each region, the total consumption is equal to the sum of wages earned by workers characterized by different skill levels and entrepreneurs’ profits. We consider three skill levels *l*: *H*-high, *M*-medium, and *L*-low. Formally, the total consumer expenditures ${b}_{kt}$ in region *k* at time *t* are equal to:

1 $$b_{k,t} = \mathop \sum \limits_{l} w_{l,k,t} n_{kl,t} + \pi_{k,t} ,$$

where ${\pi }_{k,t}$ is the sum of entrepreneurs’ profits in region *k* (discussed in the Price setting section below), while ${w}_{k,l,t}$ and ${n}_{k,l,t}$ are after-tax wages and the number of workers characterized by skill-level *l* in region *k*, respectively. The number of workers ${n}_{k,l,t}$ is computed as the sum of workers employed in all sectors in region *k* of a given skill level: ${n}_{k,l,t}={\sum }_{s}{n}_{{s}_{k},l,t}$, where ${n}_{{s}_{k},l,t}$ is the number of workers of skill *l* employed in sector* s* in region *k* at time *t.*

**Figure 4:**
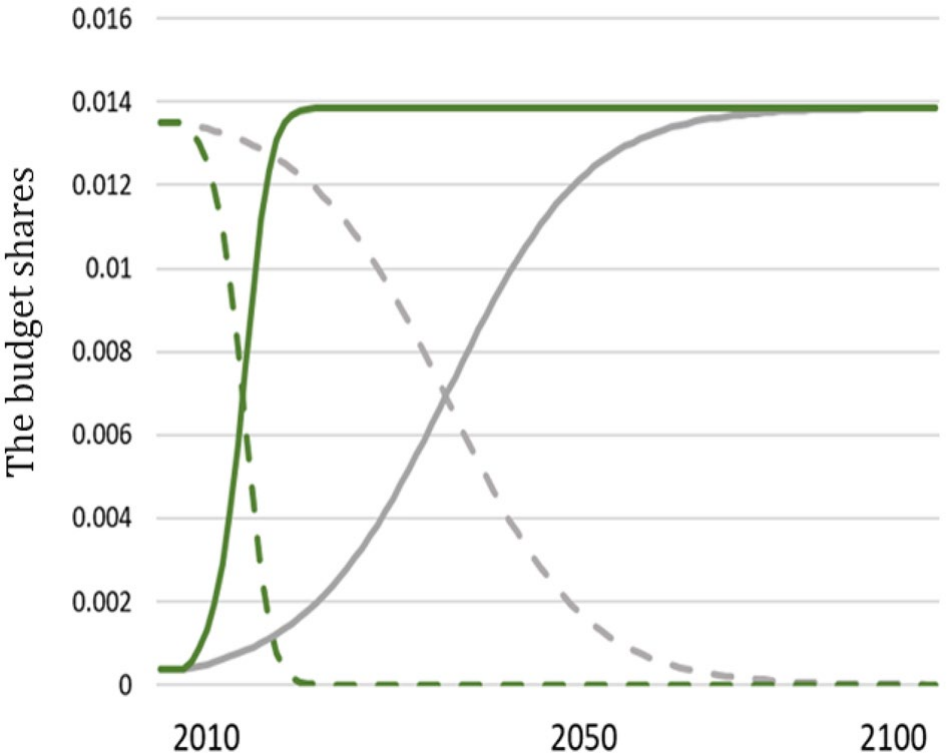
Evolving budget shares. The solid line shows the evolution of the share of budget spent on the EU goods, and dashed lines on the NEU goods, by the EU representative consumer in an exemplary sector. The total share of the budget spent on goods from the exemplary industry is constant over time. However, the share of goods purchased from the region where the sector’s s products are cheaper increases either slowly (grey lines) or rapidly (green lines) over time.

We consider the followings ‘consumption’ scenarios:

1. In the ‘constant budget shares’ scenario, we assume that consumers allocate constant shares of their budgets across goods from different industries and regions, according to the vectors of household budget shares. These shares do not change over time. The total demand in region *k* for products of sector *s* produced in region *j* is equal to: ${f}_{k,{s}_{j},t}=$ $\frac{{{b}_{k,t}*g}_{k,{s}_{j}}}{{p}_{{s}_{j},t-1}}$, where ${p}_{{s}_{j},t-1}$ is the average price of goods in sector *s* from region *j,* and ${g}_{k,{s}_{j}}$ is the share of consumer expenditures in region *k* spent on commodities from sector *s* produced in region *j*. It is important to note that according to the EXIOBASE data, consumers spend money on products of sector *s* that are produced in different regions. In the ‘constant budget’ scenario, goods produced in the same sector but in different regions are assumed to be non-substitutable.

2. In the ‘evolving budget shares’ scenario, budget shares are constant across sectors, but goods from the same industry are substitutable across regions. In particular, the total share of consumption expenditures in region *k* spent on goods from sector *s* ${(g}_{k,s})$ is constant over time. It is equal to the sum of shares spent on *s*-goods from both regions: ${g}_{k,s}={g}_{k,{s}_{EU},t}+{g}_{k,{s}_{NEU},t}$, where ${g}_{k,{s}_{EU},t}$ and ${g}_{k,{s}_{NEU},t}$ are the share of consumer expenditures in region *k* spent on commodities from sector *s* produced in the EU and in the NEU, respectively. These shares change over time according to the replicator dynamics (see Dosi et al.^50^):

2a $${\dot{g}}_{k,{s}_{EU},t}={\alpha *g}_{k,{s}_{EU},t}*\left(1-{g}_{k,{s}_{EU},t}\right)\left({\zeta }_{{s}_{EU},t}-{\zeta }_{{s}_{NEU},t}\right),$$

2b $${\dot{g}}_{k,{s}_{NEU},t}={\alpha *g}_{k,{s}_{NEU},t}*\left(1-{g}_{k,{s}_{NEU},t}\right)\left({\zeta }_{{s}_{NEU},t}-{\zeta }_{{s}_{EU},t}\right),$$

where ${\zeta }_{{s}_{EU},t}$ and ${\zeta }_{{s}_{NEU},t}$ capture the competitiveness of commodities from sector *s* in both regions. The competitiveness of the EU products from sector *s* is equal to: ${\zeta }_{s,EU,t}=\frac{\mathrm{exp}\left(-{p}_{{s}_{EU},t}\right)}{\mathrm{exp}\left(-{p}_{{s}_{EU},t}\right)+\mathrm{exp}\left(-{p}_{{s}_{NEU},t}\right)}$, while the competitiveness of the NEU products is equal to: ${\zeta }_{s,NEU,t}=\frac{\mathrm{exp}\left(-{p}_{{s}_{NEU},t}\right)}{\mathrm{exp}\left(-{p}_{{s}_{EU},t}\right)+\mathrm{exp}\left(-{p}_{{s}_{NEU},t}\right)}$, where ${p}_{{s}_{EU},t}$ and ${p}_{{s}_{NEU},t}$ are prices of sector’s *s* output in the EU and NEU, respectively. According to Eqs. 2(a) and (b), the share of cheaper products increases over time in the consumer budget. Parameter α describes the speed of such adjustments. We consider fast and slow updating: ${\alpha }_{fast}>{\alpha }_{slow}.$ Figure 4 shows the model dynamics of budget shares under fast and slow updating for the exemplary sector.

Wages change over time in the EU and NEU, according to (see Monasterolo and Raberto^51^):

3a $$\dot{w}_{EU,l,t} = - \gamma_{1EU} + \gamma_{2EU} U_{EUt} ,$$

3b $${\dot{w}}_{NEU,l,t}=-{\gamma }_{1NEU}+{\gamma }_{2NEU}{U}_{NEUt},$$

where ${U}_{EUt}$ and ${U}_{NEUt}$ are employment rates in the EU and outside, respectively, with ${\gamma }_{1k}<{\gamma }_{2k}$. The employment rates are computed as the fraction of total employment divided by workforce. Parameter ${\gamma }_{1k}$ sets the wage decrease rate when the employment rate is zero, and the ratio ${\gamma }_{1k}/{\gamma }_{2k}$ is the value of the employment rate that results in a constant wage.

We assume that wages follow the same dynamics, described by Eqns. 3a and 3b, regardless of the skill level. The numbers of workers in the EU ($${L}_{EUt})$$ and in the NEU ($${L}_{NEUt}$$) grows over time according to:

4 $${L}_{EU,t}=\left(1+{\sigma }_{EU}\right){L}_{EU,t-1}$$

5 $${L}_{NEU,t}=\left(1+{\sigma }_{NEU}\right){L}_{NEU,t-1},$$

where ${\sigma }_{EU}$ and ${\sigma }_{NEU}$ are the annual growth rates of the population in the EU and NEU, respectively.

In the baseline model simulations, we assume that ${\gamma }_{1EU}={\gamma }_{1NEU}$ and ${\gamma }_{2EU}={\gamma }_{2NEU}$. We consider two additional scenarios:

1. The ‘increasing the wage gap’ scenario, in which ${\gamma }_{2EU}>{\gamma }_{2NEU}$. This implies that the growth of wages in the EU exceeds their growth outside the EU.
2. The ‘closing the wage gap’ scenario, where ${\gamma }_{2EU}<{\gamma }_{2NEU}$ and the gap between wages in the EU and NEU decreases over time.

### Production

The output in each sector is determined as follows. In the first step, the vector of desired production $Xd$ in each sector is computed using the Leontief inverse matrix:

6 $$Xd={\left(I-A\right)}^{-1}F,$$

where *F* is the vector of final demand computed as the sum of consumer demand in the EU (*F*_*EU*_ ) and in the NEU (*F*_*NEU*_), as well as government orders in both regions. *A* is the matrix of technical coefficients of the hybrid input–output table, where each coefficient ${\alpha }_{ij}$ indicates how much input *i* is required to produce one unit of output *j*. A firm may not be able to satisfy its desired demand due to insufficient capital. If this happens, we rescale the vector of final demand considering firms’ capacity constraints (see Pichler and Farmer^52^ for the discussion of different rationing algorithms).

Formally, if the desired production in sector *s* in region *j* (${x}_{{s}_{j}}^{d}$) exceeds its maximum productive capacity ${q}_{{s}_{j},t}^{max}$, the final demand for the constrained sector is rescaled. For each sector *c* from region *j* unable to achieve its desired output, we compute a decrease (y%) in final demand as follows:

7 $$\left({m}_{{c}_{j},{1}_{EU}}{f}_{{1}_{EU}}+{m}_{{c}_{j},{2}_{EU}}{f}_{{1}_{EU}}+\dots +{m}_{{c}_{j},{1}_{NEU}}{f}_{{1}_{NEU}}+{m}_{{c}_{j},{2}_{EU}}{f}_{{1}_{NEU}}+..\right)\left(1-{\mathrm{y}}_{{c}_{j}}\mathrm{\%}\right)={q}_{{c}_{j}}^{max}$$

8 $${\mathrm{y}}_{{c}_{j}}\mathrm{\%}=1- \frac{{q}_{{c}_{j}}^{max}}{{m}_{{c}_{j},{1}_{EU}}{f}_{{1}_{EU}}+{m}_{{c}_{j},{2}_{EU}}{f}_{{1}_{EU}}+\dots +{m}_{{c}_{j},{1}_{NEU}}{f}_{{1}_{NEU}}+{m}_{{c}_{j},{2}_{EU}}{f}_{{1}_{NEU}}+..}$$

where ${c}_{j}$ stands for the constrained sector, $F$ is the vector of final demand, which entries define the sum of final demand from the EU and NEU, e.g., ${f}_{{1}_{EU}}$ and ${f}_{{1}_{NEU}}$ describe the sum of final demand from both regions for goods from sector 1 produced in the EU and NEU, respectively. Finally, ${m}_{ij}$ are entries of matrix *M* computed as the Leontief Inverse of matrix *A*: $$M={\left(I-A\right)}^{-1}=\left[\begin{array}{ccc}{m}_{{1}_{EU},{1}_{EU}}& {m}_{{1}_{EU},{2}_{EU}}& ..\\ {m}_{{2}_{EU},{1}_{EU}}& {m}_{{2}_{EU},{2}_{EU}}& ..\\ ..& ..& ..\end{array}\right]$$.

The new vector of final demand ${F}_{new}$ that satisfies the condition ${F}_{new}\le {Q}_{{c}_{j}}^{max}$ is calculated as:

9 $${F}_{new}=\left(\begin{array}{c}{f}_{{1}_{EU}}\\ {f}_{{2}_{EU}}\\ \dots \\ {f}_{{1}_{NEU}}\\ \dots \end{array}\right)\left(1-y\%\right),$$

where y% is a scalar, computed as the maximum of ${\mathrm{y}}_{{c}_{j} }\mathrm{\%}$ among all constrained sectors. Once ${F}_{new}$ is computed, we calculate the vector of actual production according to: $X={\left(I-A\right)}^{-1}{F}_{new}$.

### ABM: Capital sector and innovation

Our work differs from the previous input–output studies in that we add a vector ${Q}_{max}$ to the IOT (Fig. 3). Its entries ${q}_{{s}_{j}}^{max}$ define the maximum output that sector *s* in region *j* can produce at time *t*. The capital stock held by a firm-sector *s* in region *j* defines its maximum output, which can be computed as:

10 $${q}_{{s}_{j},t}^{max}={\sum }_{g}{A}_{g}{k}_{g,{s}_{j}t},$$

where ${k}_{{s}_{j},t}$ is the stock of vintage capital *g* owned by a representative firm-sector *s* in region *j* at time* t*, and ${A}_{g}$ is the productivity of vintage *g.* Each capital stock depreciates over time at a rate of *δ*: ${\mathrm{k}}_{g,{s}_{j},\mathrm{t}}=(1-\updelta ){\mathrm{k}}_{g{,s}_{j},\mathrm{t}-1}$. The right-hand side of Eq. (10) sums up all vintages of capital *g* owned by firm-sector *s* in region *j.* A firm that acquires a capital stock characterized by productivity ${A}_{g}$ can produce more goods than a firm owning the same level of capital stock but characterized by a lower than $g$ productivity.

In case a firm in sector *s* cannot produce the desired output because of insufficient capital (${q}_{{s}_{j},t}^{max}<{x}_{{s}_{j},t}^{d}$), it orders capital on the capital market. The amount of capital ordered by firm *s* in sector *j* is equal to:

11 $$K_{{order, s_{j} ,t}} = \left( {1 + \xi } \right)*\left( {\frac{{x_{{s_{j} ,t}}^{d} - q_{{s_{j} ,t}}^{max} }}{{A_{i} }} } \right) \quad {\text{if}}\quad x_{{s_{j} ,t}}^{d} - q_{{s_{j} ,t}}^{max} > 0,$$

where ${A}_{i}$ is the capital productivity of the capital good, the supplier of which is chosen with a probability proportional to its competitiveness, $\xi$ captures capital investment above necessary capital expansion to account for the capital depreciation in the next period, ${x}_{{s}_{j},t}^{d}$ is the desired production level of a firm-sector *s* in region *j* at time *t*.

The capital sector consists of heterogeneous firms producing capital goods using techniques that differ in terms of labor productivity. In particular, each capital firm *i* is characterized by the productivity of low (${\alpha }_{Lit}$), medium (${\alpha }_{Mit})$ and high-${(\alpha }_{Hit})$ skilled workers, as well as the productivity of the capital that it offers for sale (*A*_*i*_). The unit cost of firm *i* is equal to: ${c}_{it}={\sum }_{l}{\alpha }_{li}{w}_{l,k,t}+\sum_{s,j}{\alpha }_{M{s}_{j}}{p}_{{s}_{j},t}$ with ${\alpha }_{l}$ and ${w}_{l,k,t}$ capturing the productivity and wages of workers of different skills in region *k*: *l* = {*M,H,L*}. Capital firms can operate in the EU or in the NEU, which affects their labor costs. Firms are assigned their location at random in the initial step of model simulations. The last component of the cost equation $\sum_{s,j}{\alpha }_{M{s}_{j}}{p}_{{s}_{j},t}$ captures unit expenditures of capital firms on inputs from other sectors. These are represented by a vector corresponding to the 'Manufacture of machinery and equipment’ in the EXIOBASE database.

A firm from sector *s*, which wants to invest in capital expansion, chooses capital firm *i* at random with a probability proportional to its competitiveness. The competitiveness is defined as: $\frac{{c1}_{it}}{{A}_{it}}$, where ${c1}_{it}={\sum }_{l}{\alpha }_{li}{w}_{l,k,t}$ is the unit cost of labor for the firm *i*. We use the unit cost of labor instead of the total unit cost as the measure of competitiveness. This is motivated by the fact that capital firms are characterized by the same technical coefficients describing input use from different sectors, in line with the EXIOBASE database. Thus, they compete by improving labor productivities. This works as follows. Each capital firm collects capital orders from different sectors. The sum of all orders defines the final demand for capital in the vector of final demand* F*. This allows the computation of total inputs from other sectors that are delivered to the capital sector. We assume that households do not order capital, and all demand comes from firms. If the supply of inputs is insufficient to satisfy the demand of capital firms, the production of each capital firm is reduced, i.e., the inputs are distributed among different capital firms in proportion to their demand. In this case, firms in different sectors would receive less capital than they have ordered.

Innovation in the capital sector is modelled as a two-stage process^50, 51^. First, firms draw if they engage in innovation. Second, they sample productivity improvements. In our model, we consider two types of innovations: with probability ${p}_{innov1}$, a firm improves its labor productivity, while with probability ${p}_{innov2}$, a firm improves the productivity of capital offered to its clients. First, a capital firm chooses the type of labor productivity (skill-level) it wants to improve. In particular, firms aim at improving the productivity of labor, which constitutes the highest share of their labor expenditures. If successful, the firm draws a new labor productivity from the range of its own productivity and the maximum productivity in the market. When a firm improves the productivity of capital, it draws its new productivity from between its own productivity and the maximum productivity frontier ${A}_{max,t}$. The latter increases over time at the rate σ to reflect general progress: ${A}_{max,t}=(1+\upsigma ){A}_{max,t-1}$. Each time step, a new capital firms enters the market with the probability ${p}_{new}$. It receives a loan from the bank ${l}_{jt}$ to cover initial input expenses, which has to be repaid after τ periods. In the event that a firm is unable to do this, or its deposits become negative, it goes bankrupt.

### Price setting

After each sector decides on the production and investment in capital expansion, the vector of final prices is computed, according to: $P=(I-{A}_{m}\left(1+\mu \right){)}^{-1}EX*(1+\mu )$, where $\mu$ is the markup, which we assume is constant and homogenous across sectors. Markup determines profits in each sector (${\pi }_{{s}_{j},t})$, i.e., earnings above the production costs. The sum of entrepreneurs’ profits in each region (${\pi }_{j,t}={\sum }_{j}{\pi }_{{s}_{j},t}$) is distributed as dividends to consumers in each region (see Eq. 1).

The vector *EX* captures costs of capital amortization, services, and labor expenses:

12 $$ex_{{s_{j} ,t}} = \mathop \sum \limits_{l} \alpha_{{ls_{j} }} w_{j,l,t} + \frac{{\mathop \sum \nolimits_{g} \delta k_{{g,s_{j} ,t}} }}{{x_{{s_{j} ,t}} }} + \mathop \sum \limits_{m} a_{{s_{j} m}} .$$

where ${\sum }_{l}{\alpha }_{l{s}_{j}}{w}_{j,l,t}$ are spending on wages by sector ${s}_{j}$; ${\sum }_{g}\delta {*k}_{g,{s}_{j},t}$ is capital amortization and $\sum_{m}{a}_{{s}_{j}m}$ are service expenditures per unit of output on sector-services *m*. It is important to note that the matrix of technical coefficients ${A}_{m}$ is the same as matrix *A* with one exception: technical coefficients corresponding to services in matrix *A* are set to 0. This is motivated by the fact that the hybrid EXIOBASE database includes the coefficients expressed both as volumes and values, which explains the necessity of such an adjustment. In particular, all coefficients ${a}_{mij}$ of matrix ${A}_{m}$ define the tons of input of industry *i* needed to produce one ton of output in industry *j*. The coefficients ${a}_{ij}$ in matrix *A*, which are set to 0 in matrix ${A}_{m}$, describe the number of millions (Euro) needed to produce one ton of output.

### The circular economy

The EXIOBASE database includes data on the technical coefficients of primary and secondary production for the following sectors: wood, pulp, paper, plastic, glass, steel, precious metals, aluminum, ‘lead, zinc and tin’, copper, other non-ferrous metals, and construction. For instance, aluminum can be produced from aluminum ore (primary production) or from aluminum scrap (secondary production). The EXIOBASE database specifies technical coefficients of both sectors.

In our model, primary *s* and secondary *si* production are assumed to be perfect substitutes sold at one price. A firm can produce output using both techniques. Every period, a firm decides how much of its output to produce in the secondary sector. To avoid the simultaneity problem, we assume that secondary production is not available until the next period, while primary output is produced on the spot. The stock of secondary production from the previous period is used to satisfy the demand in the next period, reducing firms’ desired production. The simultaneity problem arises because the decision of how much output to produce with secondary technology depends on the relative unit costs of primary and secondary production. In turn, the relative unit cost of both technologies depends on how much output is produced with each of these technologies as this affects production and prices in all sectors of the IO table.

Formally, the desired production of secondary input *si* in region *j* is equal to:

13 $$f_{{si_{j} }} = {\text{min}}\left( {0.85*x_{{s_{j} }}^{e} ,q_{{si_{j} ,t}}^{max} } \right)\quad {\text{if}}\quad c_{{s_{j} ,t}} > c_{{si_{j} ,t}}$$

14 $$f_{{si_{j} }} = {\text{min}}\left( {x_{{s_{j} }}^{e} - q_{{s_{j} ,t}}^{max} ,q_{{si_{j} ,t}}^{max} } \right)\quad {\text{otherwise}},$$

where ${x}_{{s}_{j}}^{e}$ is equal to the expected production in the next period; ${q}_{{si}_{j},t}^{max}$ is the maximum production capacity of the secondary sector; ${c}_{{s}_{j},t}$ and ${c}_{{si}_{j},t}$ are unit costs of production of primary and secondary technology, respectively. If secondary production is characterized by a higher unit cost than primary production, a firm will only produce its excess demand (above the plant’s production capacity) using the former technique: ${x}_{{s}_{j}}^{e}-{q}_{{s}_{j},t}^{max}$. The desired secondary production enters the vector of final demand ${f}_{{si}_{j}}$.

We assume that even if the secondary production is cheaper, a firm wants to invest in capital to be able to produce 15% of its output using the primary production technique, which explains 0.85 in Eq. (13) and 0.15 in Eq. (17).There are two reasons behind this assumption. First, many materials lose their quality during recycling, which places limits on recycling^53, 54^, and questions about whether achieving 100% recycling is possible^55^. In many industries, raw materials are mixed with scrap to produce recycled materials. For example, primary steel is mixed with scrap steel to produce secondary steel^56^. Second, in model simulations without the "0.15 rule," scrap shortages often led to a sudden decline in economic output. This is because in a growing economy, the amount of scrap is insufficient to support 100% production from recycled materials, and an inflow of virgin materials is required. We chose a value of 15% because initial model simulations have shown that this is the smallest value sufficient to prevent an economic downturn due to scrap shortages. All in all, the assumption that firms maintain the production capacity of the primary sector at 15% allows them to respond to fluctuations in scrap availability. Calibrating our model on a different growth pattern (than that currently assumed) would require recalibrating this value.

There is a possibility that the demand for scrap in sector *s* from both regions exceeds its supply. In this case, scrap is allocated between secondary sectors in the EU and in the NEU in proportion to their demand. The amount of scrap in sector *s* evolves over time: it is reduced by demand for scrap ${d}_{{s}_{j},t}$ from sector* s* in both regions, while it increases depending on household consumption and production:

15 $$scrap_{s,t} = scrap_{s,t - 1} - \mathop \sum \limits_{j} d_{{s_{j,t} }} + \mathop \sum \limits_{j} A1X_{{s_{j} ,t - 1}} + \mathop \sum \limits_{j} A2F_{{s_{j} ,t - 1}}$$

where *A1* and *A2* are vectors of coefficients describing how much waste (scrap) *s* each sector produces from total production and consumption, respectively. The coefficients were calibrated using the EXIOBASE environmental accounts.

An important property of our model that distinguishes it from the preceding work is that firms decide whether to expand capital in the primary or secondary sector based on their unit costs. If the unit cost of production of the secondary product is cheaper than for the production of the primary product, a firm invests in capital expansion of the secondary sector. In particular, its capital investment in the secondary sector is equal to:

16 $$K_{{order, si_{j,} t}} = \left( {1 + \xi } \right)*\left( {\frac{{0.85*x_{{s_{j} ,t}} - q_{{si_{j} ,t}}^{max} }}{{A_{i} }}} \right),\quad {\text{if}}\quad c_{{s_{j} ,t}} > c_{{s_{ij} ,t}} ,$$

where ${A}_{i}$ is productivity of a new capital good, $\xi$ captures capital investment above necessary capital expansion, ${c}_{{s}_{j},t}$ and ${c}_{{si}_{j},t}$ are the unit costs of primary and secondary production in sector *s*, region *j*, at time *t.* The firm also invests in the capital expansion of the primary sector so as to be able to produce 15% of the desired production therein:

17 $$K_{{order, s_{j} t}} = \left( {1 + \xi } \right)*\left( {\frac{{0.15*x_{{s_{j} ,t}} - q_{{si_{j} t}}^{max} }}{{A_{i} }}} \right),\quad {\text{if}}\quad c_{{s_{j} ,t}} > c_{{s_{ij} ,t}} .$$

If the primary production is cheaper than the secondary production, a firm does not invest in the recycling infrastructure and the primary sector is treated as all other sectors in an input–output analysis.

In sectors that produce output using two techniques, the price of its final output is described by:

18 $$p_{{s_{j} t}} = \left( {1 + \mu } \right)\left( {\mathop \sum \limits_{g} a_{{g,s_{j} }} p_{{g,s_{j} ,t}} + ex_{{s_{j} }} } \right)\frac{{x_{{s_{j} ,t}} }}{{x_{{s_{j} ,t}} + x_{{si_{j} t, - 1}} }} + p_{{si_{j} ,t - 1}} \frac{{x_{{si_{j} ,t - 1}} }}{{x_{{s_{j} ,t}} + x_{{si_{j} t, - 1}} }}.$$

where $\upmu$ is the markup; ${x}_{{si}_{j},t}$ and ${x}_{{s}_{j},t}$ are production levels using secondary and primary inputs, respectively; ${p}_{{s}_{j},t}$ is the price of primary production, ${p}_{{s}_{ij},t}$ is the price of secondary production, while ${\sum }_{g}{{a}_{g{s}_{j}}p}_{g{,s}_{j},t}$ captures the cost of all inputs used in the primary production. According to Eq. (18), the price of the final output is the average of primary and secondary production weighted by their shares in the total production. If ${x}_{{si}_{j},t}$=0, the price of sector *s* reduces to the price of primary product.

### Supplementary Information

Supplementary Information.

## Data Availability

The datasets/codes used in the current study are available at https://osf.io/3vdpr/.

## References

[1] Krausmann F, Gingrich S, Eisenmenger N, Karl-Heinz E, Haberl H, Fischer-Kowalski M (2009). Growth in global materials use, GDP and population during the 20th century. Ecol. Econ..

[2] Sovacool BK, Ali SH, Bazilian M, Radley B, Nemery B, Okatz J (2020). Sustainable minerals and metals for a low-carbon future. Science.

[3] Manberger A, Stenqvist B (2018). Global metal flows in the renewable energy transition: exploring the effects of substitutes, technological mix and development. Energy Policy.

[4] IEA (2021). The Role of Critical Minerals in Clean Energy Transitions.

[5] Massari S, Ruberti M (2013). Rare earth elements as critical raw materials: Focus on international markets and future strategies. Resources Policy.

[6] Michaux, S. The Mining of Minerals and the Limits to Growth. Geological Survey of Finland Report (2021).

[7] Nassar NT, Graedel TE, Harper EM (2015). By-product metals are technologically essential but have problematic supply. Sci. Adv..

[8] McKinsey 2016. The circular economy: moving from theory to practice. Downloaded at: www.mckinsey.com

[9] EU, 2020. A New Circular Economy Action Plan. COM/2020/98.

[10] Kirchherr J, Reike D, Hekkert M (2017). Conceptualizing the circular economy: an analysis of 114 definitions. Resources Conserv. Recycl..

[11] Korhonen J, Honkasalo A, Seppala J (2018). Circular economy: the concepts and its limitations. Ecol. Econ..

[12] Morseletto P (2020). Targets for a circular economy. Resource Conserv. Recycl..

[13] Milios, L. Policies for Resource Efficient and Effective Solutions: A Review of Concepts, Current Policy Landscape and Future Policy Considerations for the Transition to a Circular Economy. Mistra REES Report (2016).

[14] Ranta V, Aarikka-Stenroos L, Ritala P, Mäkinen SJ (2018). Exploring institutional drivers and barriers of the circular economy: a cross-regional comparison of China, the US, and Europe. Resource Conserv. Recycl..

[15] Towa E, Zeller V, Achten WMJ (2020). Input-output models and waste management analysis: A critical review. J. Cleaner Product..

[16] Prendeville, S., Sanders, C., Sherry, J., & Costa, F. Circular economy: is it enough? Ecocenter Design Technical Report (2014).

[17] Urbinati A, Franzò S, Chiaroni D (2021). Enablers and Barriers for Circular Business Models: An empirical analysis in the Italian automotive industry. Sustain. Prod. Consump..

[18] Zinc T, Geyer R (2017). Circular Economy rebound. J. Ind. Ecol..

[19] Castro CG, Hofman Trevisan A, Pigosso DCA, Mascarenhas J (2020). The rebound effect of circular economy: definitions, mechanisms and a research agenda. J. Cleaner Product..

[20] Babiker MH (2005). Climate change policy, market structure and carbon leakage. J. Int. Econ..

[21] Tisserant A, Pauliuk S, Merciai S, Schmidt J, Fry J, Wood R, Tukker A (2017). Solid waste and the circular economy: a global analysis of waste treatment and waste footprint. J. Ind. Ecol..

[22] Giljum S, Bruckner M, Martinez A (2014). Material footprint assessment in a global input-output framework. J. Ind. Ecol..

[23] Wiebe KS, Harsdorff M, Montt G, Simas MS, Wood R (2019). Global circular economy scenarios in a multiregional input-output framework. Environ. Sci. Technol..

[24] Donati F, Niccolson S, de Koning A, Daniels B, Christis M, Boonen K, Geerken T, Rodrigues JFD, Tukker A (2020). Modelling the circular economy in environmentally extended input-output: a web application. J. Ind. Econ..

[25] Yamamoto T, Meciai S, Mogollon JM, Tukker A (2022). The role of recycling in alleviating supply chain risk -insights from a stock flow consistent perspective using a hybrid input-output database. Resources Conserv. Recycl..

[26] Towa E, Zeller V, Achten WMJ (2021). Using a multiregional hybrid input-output model: The case of Belgium and its regions. Sustain. Prod. Consum..

[27] Cimpan C, Bjelle EL, Stromman AH (2021). Plastic packaging flows in Europe: a hybrid input-output approach. J. Ind. Ecol..

[28] Aguilar-Hernandez GA, Sigüenza-Sanchez CP, Donati F, Rodrigues JFD, Tukker A (2018). Assessing circularity interventions: A review of EEIOA-based studies. J. Econ. Struct..

[29] Tan X, Liu Y, Cui J, Su B (2018). Assessment of carbon leakage by channels: an approach combing CGE model and decomposition analysis. Energy Econ..

[30] Axtell, R., & Farmer, D. Agent-based modelling in economic and finance: past, present, and future. J. Econ. Lit. (forthcoming) (2022).

[31] Cincotti S, Raberto M, Teglio A (2022). Why do we need agent-based macroeconomics?. Rev. Evolut. Polit. Econ..

[32] Franke R, Westerhoff F (2012). Structural stochastic volatility in asset pricing dynamics: estimation and model contest. J. Econ. Dyn. Control.

[33] Thurner S, Polenda S (2013). Debrank-transparency: controlling systemic risk in financial networks. Sci. Rep..

[34] Dosi G, Fagiolo G, Roventini A (2010). Schumpeter meeting Keynes: a policy-friendly model of endogenous growth and business cycles. J. Econ. Dyn. Control.

[35] Russo A, Riccetti L, Gallegti M (2016). Increasing inequality, consumer credit and financial fragility in an agent based macroeconomic model. J. Evolut. Econ..

[36] Mazzocchetti A, Raberto M, Teglio A, Cincotti S (2018). Securitization and business cycle: An agent-based perspective. Ind. Corp. Change.

[37] Botta A, Caverzasi E, Russo A, Gallegati M, Stiglitz J (2021). Inequality and finance in a rent economy. J. Econ. Behav. Org..

[38] Lamperti F, Bosetti V, Roventini A, Tavoni M (2019). The public costs of climate-induced financial instability. Nat. Clim. Change.

[39] Safarzynska K, van den Bergh J (2022). ABM-IAM: optimal climate policy under bounded rationality and multiple inequalities. Environ. Res. Lett..

[40] Poledna S, Miess MG, Hommes C, Rabitsch K (2022). Economic forecasting with an agent-based model. Eur. Econ. Rev..

[41] Niamir L, Filatova T, Voinov A, Bressers H (2018). Transition to low-carbon economy: Assessing cumulative impacts of individual behavioral changes. Energy Policy.

[42] Leimbach M, Kriegler E, Roming N, Schwanitz J (2017). Futures growth patterns of world regions—A GDP scenario approach. Global Environ. Change.

[43] Hofbauer J, Sigmund K (2003). Evolutionary game dynamics. Bull. Am. Math. Soc..

[44] Magrini S, Vernon-Henderson J, Thisse J-F (2004). Regional (di)convergence. Handbook of Regional and Urban Economics.

[45] Hund, K., la Porta, D., Fabregas, T.P., Laing, T., & Drexhage, J. Minerals for Climate Action: The mineral intensity of the clean energy transition. World Bank Report (2020).

[46] Elshkaki A, Graedel TE, Ciacci L, Reck BK (2016). Copper demand, supply and associated energy use to 2050. Global Environ. Change.

[47] Schipper BW, Lin H-C, Meloni MA, Wansleeben K, Heijungs R, Can-der-Voet E (2018). Estimating global copper demand until 2100 with regression and stock dynamics. Resource Conserv. Recycl..

[48] Nelson RR, Winter SG (1982). An Evolutionary Theory of Economic Change.

[49] Dosi G, Fagiolo G, Napoletano M, Roventini A (2013). Income distribution, credit and fiscal policies in an agent-based Keynesian model. J. Econ. Dyn. Control..

[50] Dosi G, Roventini A, Russo E (2019). Endogenous growth and global divergence in a multi-country agent-based model. J. Econ. Dyn. Control.

[51] Monasterolo I, Raberto M (2018). The EIRIN flow-of-funds behavioural model of green fiscal policies and green sovereign bonds. Ecol. Econ..

[52] Pichler, A., & Farmer, J.D. Modeling simultaneous supply and demand shocks in input-output networks. INET Oxford Working Paper No. 2021-05 (2021).

[53] Sandin G, Peters G (2018). Environmental impact of textile reuse and recycling a review. J. Clean. Prod..

[54] Safarzynska K, Di Domenico L, Raberto M (2023). Circular economy mitigates the material rebound due to investments in renewable energy. J. Clean Prod..

[55] Parrique T., Barth J., Briens F., C. Kerschner, Kraus-Polk A., Kuokkanen A., & Spangenberg J. H. Decoupling debunked: Evidence and arguments against green growth as a sole strategy for sustainability. European Environmental Bureau (2019).

[56] Winning M, Calzadilla A, Bleischwitz R, Nechifor V (2017). Towards a circular economy: insights based on the development of the global ENGAGE-materials model and evidence for the iron and steel industry. Int. Econ. Econ. Policy.

