# Supplementary material for: The leakage effect may undermine the circular economy efforts
Source: Sci Rep. 2023 Oct 4;13:16677. doi: 10.1038/s41598-023-44004-x (PMC10550933; doi:10.1038/s41598-023-44004-x)
Supplement: Supplementary file 1 — Supplementary Information. [file 41598_2023_44004_MOESM1_ESM.docx]

**Supplementary Materials**

**Calibration**

In the paper, we use the hybrid EXIOBASE database for 2011, which is a multiregional input-output table (MRIO), describing interregional and intersectoral material flows. The database specifies tons of inputs from each sector needed to produce a ton of output in a given industry. There are exceptions: some inputs are measured in million Euros, where an output of the industry is a service, or terra joules (TJ) for the electricity sector. The database includes 44 countries and 5 aggregated regions. For our purposes, we aggregated the data into two regions: the EU and the rest of the world (NEU). This has been done by summing the volume of each input used in a given industry in European countries and the rest of the world. To compute technical coefficients of matrix *A*, where each entry *a_ij_* describes how many tons of input *i* is used by industry *j* to produce a unit of its output, we divided the total input use by the total volume (tons) of production in each region.

The EXIOBASE database provides a detailed account of flows between sectors, but not stocks. As a result, it ignores capital accumulation, which is one of the main drivers of economic growth. As the detailed data on capital are not available, we calibrated the initial level of capital in each sector as follows. Initial capital $K_{s0}$and capital intensity $\varphi_{s}$of each sector s are calibrated to be equal in 2011 to (see Yilmaz and Kilic, 2021):

$K_{s0}=\frac{I_{s0}}{\delta+g_{s}}$, (20)

$\varphi_{s}=\frac{K_{so}}{q_{so}}$. (21)

where $g_{s}$ is the annual growth of production of sector s between 2010 and 2011, which we calculated using the monetary EXIOBASE for both years, while $I_{s0}$ are investments in capital (machinery) by sector *s* in 2011. The hybrid EXIOBASE database contains additional data on: labour and waste, which we used to calibrate the following parameters. In particular, we aggregated total employment in each sector across countries in the EU and in the rest of the world to calculate the number of persons needed per a unit of output. This was done separately for different skill levels. In addition, we aggregated data on waste demand and supply (stocks of scrap) by each sector and final consumption. The shares of budget spent by consumers in the EU and in the NEU as well as government orders in each sector were calibrated using the monetary EXIOBASE database.

We calibrated the initial level of workforce in the EU in 2011 and the growth of population in the EU between 2011 and 2020 using data from the EUROSTAT, while for the rest of the world using the Word Bank database. The initial workforce in the EU in 2011 was equal to 166 million people, while globally it was 3.1 billion people. As the input-output database does not consider employment in the public sector, which constitutes about 1/3 of total employment in the EU and 1/2 globally, we set workforce available for work in industrial sectors to be equal to 107 million in the EU and 1.55 billion globally. The growth rate of workforce in the EU $\sigma_{EU}$ was calibrated based on the expect growth of population in the EU between 2011 and 2100. During this time, the EU population is expected to increase from 501 to 608 million, while the global population from 6.9 to 10.4 billion people, which implies $\sigma_{EU}=0.00218$ and $\sigma_{NEU}=0.00475$.

The productivity of initial capital stock is set equal to 2.5 in all sectors, while we set the depreciation rate equal to 10%, and the annual growth of the productivity frontier to σ=0.05. These values were chosen so that the rate of growth of consumption per capita between 2010 and 2100 is equal on average to 1.6% for δ=10%, which is consistent with the SSP1 growth scenario (Leimbach et al., 2017).

On the labour market, we set parameters in the wage equations a $\gamma_{1EU}=\gamma_{1EU}=0$and $\gamma_{2EU}=\gamma_{2NEU}=0.5\%$. We modify these parameters in the ‘increasing the wage gap’ scenario to be equal to $\gamma_{1EU}$=1% and $\gamma_{1NEU}=0.5\%$, while in the “closing the wage gap” scenario as $\gamma_{1EU}$=0.5% and $\gamma_{1NEU}=1\%$. If not specified otherwise, other parameters were chosen from realistic values to ensure that dynamics of macroeconomic variables such as economic output and consumption replicate the expected patterns of SSP1 projections.

**References**

Yilmaz, E., Kilic, I., 2021. Estimating firm-level capital stock: the evidence from Turkey. The Developing Economies 59: 371-404.

Leimbach M, Kriegler, E., Roming, N., Schwanitz, J., 2017. Futures growth patterns of world regions - a GDP scenario approach Global Environmental Change 42: 215–25.

*Supplementary Table 1. The overview of EXIOBASE tables and vectors*

| **Table/vector** | **The description** |
| --- | --- |
| *G_k_* | The vector of household budget shares |
| A | The technical coefficient matrix |
| F | The vector of final demand |
| M | The Leontief Inverse (of Matrix A) |
| X | The vector of production |
| Xd | The vector of desired production |
| $Q^{max}$ | The vector of maximum production |
| $A_{m}$ | The matrix of technical coefficients A with entries corresponding to services set to 0 |
| $EX$ | The vector *EX* captures costs of capital amortization, services and labor expenses |
| $A_{1},A_{2}$ | The vectors of coefficients describing how much each sector produces waste (scrap) from total production ($A_{1}$) and consumption ($A_{2})$ |
| P | The vector of prices |
| $N_{s_{j},l,t}$ | The vector describing the number of workers of skill *l* in region *j* employed in each secto*r s*  (per ton of output produced)*; l* refers to three skill-levels: L - low, H - high, M - medium |

*Supplementary Table 2. The overview of variables*

| **Variables** | **The description** |
| --- | --- |
| $p_{s_{j}t}$ | The price of output of sector *s* in region *j* |
| $\pi_{s_{j},t}$ | Sector’s *s* in region *j* competitiveness |
| $c_{s_{j},t}$ | Unit cost of primary production (s) |
| $c_{{si}_{j},t}$ | Unit cost of secondary production (si) |
| $c_{it}$ | Unit cost of capital firm i |
| $d_{s_{j}t}$ | Demand for scrap used in *s* in region *j* |
| ${scrap}_{s,t}$ | Stock of scrap corresponding to sector *s* |
| $K_{order, s_{j}, t}$ | Capital orders by a firm in sector *s*, region *j* at time t |
| y% | The rescaling factor |
| $\alpha_{li}$ | The productivity of labor of capital firm *i* characterized by skill-level *l* |
| $k_{g,s_{j},t}$ | Capital stock of vintage *g* by sector *s* in region *j* |
| $U_{jt}$ | Unemployment rate in region *j* |
| $L_{jt}$ | The number of employed persons in in region *j* |
| $\pi_{jt}$ | Profits of entrepreneurs in region *j* |
| $b_{jt}$ | Total consumer expenditures in region *j* |
| $\zeta_{s_{EU},t}$, $\zeta_{s_{NEU},t}$ | Competitiveness of sector *s* in the EU and NEU |

*Note:* *s, si* are subscripts indicating primary and secondary production

*Supplementary Table 3. The overview of parameters*

| Parameter/  Variable | The description | The baseline value/ or initial value |
| --- | --- | --- |
| $w_{klt}$ | The wages of works of skill *l* in region *k* | Annual wages in million Euros (initial values):  $w_{EU,H,0}$=0.11  $w_{EU,M,0}$=0.066  $w_{EU,L,0}=0.054$  $w_{NEU,H,0}=0.0418$  $w_{NEU,M,0}$=0.02112  $w_{NEU,L,0}=0.01296$ |
| $\alpha$ | Speed of adjustment of budget shares | $\alpha_{fast}$=500  $\alpha_{slow}$=100 |
| $\gamma_{1k}$ | Money-wage setting parameter (1) | 0 |
| $\gamma_{2k}$ | Money-wage setting parameter (2) | 0.005 |
| $\sigma_{k}$ | Growth of workforce in region *k* | $\sigma_{EU}$=$0.00218$  $\sigma_{NEU}$=$0.00475$ |
| $A_{g}$ | The productivity of vintage *g* | The initial productivity of capital for all firms is set to 2.5 |
| δ | Depreciation parameter | 0.1 |
| σ | the annual growth of the productivity frontier | 0.05 |
| $p_{innov1}$ | The probability that a capital firm improves own labor productivity | 0.05 |
| $p_{innov2}$ | The probability that a capital firm improves the productivity of capital offered to its clients | 0.1 |
| $p_{new}$ | Probability of a new capital firm entering the market | 0.25 |
| $\mu$ | markup | 0.2 |
| $l_{it}$ | a loan from the bank to cover initial input expenses by a capital firm *i* | 10 (thousand Euro) |
| τ | The number of periods after which the loan needs to be repaid | 10 |
| ξ | Parameter in the capital expansion function | 0.1 |

*Supplementary Table 4.* Resource use in different scenarios **(**M tons/ M TeraJoules/ Billion Euros)

|  | No CE | CE EU only | CE EU only;  Slow budget updating | CE EU only;  Fast budget  updating | Global  CE - 50% | Global CE | Global CE;  Slow budget  updating | Global CE;  Fast  budget updating |
| --- | --- | --- | --- | --- | --- | --- | --- | --- |
| Aluminum | 34.24 (2.06) | 31.43 (2.46) | 33.98 (2.02) | 31.16 (2.75) | 27.62 (1.86) | 11.22 (1.09) | 10.84 (0.97) | 8.82  (0.64) |
| Copper | 15.06 (0.91) | 13.99 (1.23) | 30.45 (2.04) | 27.49 (2.66) | 13.48 (0.92) | 9.79  (1.09) | 23.94 (2.27) | 19.02 (1.47) |
| Iron | 666.68 (39.03) | 575.47 (43.98) | 614.06 (35.7) | 560.21 (47.93) | 512.8 (33.15) | 172.37 (16.88) | 186.64 (17.29) | 147.05 (12.01) |
| Lead, zinc, tin | 9.35  (0.57) | 8.55  (0.67) | 11.19 (0.71) | 14.03 (1.33) | 8.57 (0.59) | 6.18  (0.6) | 8.06  (0.77) | 9.58  (0.75) |
| Non-ferrous  metals | 14.4  (0.86) | 13.8  (1.06) | 15.05 (0.89) | 14.64 (1.29) | 11.26 (0.74) | 3.6  (0.34) | 3.9  (0.36) | 3.83  (0.28) |
| Forest | 1274.25 (75.96) | 1190.32 (91.66) | 1278.69 (75.46) | 1182.62 (103.36) | 1177.27 (79.4) | 963.02 (89.36) | 935.86 (79.89) | 765.54 (52.85) |
| Coal | 6136.02 (363.58) | 5911.95 (454.75) | 6266.18 (371.83) | 5757.24 (508.66) | 6299.64 (428.41) | 6140.2 (559.26) | 5988.48 (505.61) | 4884.39 (338.12) |
| Petroleum | 5841.3 (366.43) | 5605.93 (454.44) | 5842.88 (363.01) | 5337.59 (490.59) | 5992.38 (429.09) | 5834.02 (563.76) | 5574.19 (496.37) | 4501.53 (323.92) |
| Gas | 3595.48 (221.14) | 3432.53 (272.2) | 3585.42 (217.89) | 3301.96 (296.62) | 3643.98 (255.42) | 3467.34 (328.29) | 3320.03 (289.37) | 2705.12 (190.34) |
| Electricity EU | 9.57  (0.47) | 9.37  (0.6) | 10.65  (0.6) | 9.7  (0.82) | 9.77 (0.55) | 9.68  (0.74) | 9.98  (0.79) | 8.16  (0.53) |
| Electricity NEU | 37.84 (2.39) | 36.19 (2.94) | 37.31 (2.28) | 34.11 (3.07) | 38.44 (2.77) | 36.73 (3.56) | 34.76 (3.06) | 28.1  (1.99) |
| Global spending | 51.23 (3.07) | 49.24 (3.74) | 50.15 (2.87) | 45.62 (3.74) | \| 52.15 (3.54) \| \| --- \| | 50.31 (4.53) | 47.35 (3.83) | 38.89 (2.42) |
| Budget EU | 10.5  (0.47) | 10.26  (0.6) | 11.71 (0.61) | 10.65 (0.81) | 10.75 (0.55) | 10.46 (0.73) | 10.9  (0.78) | 9.08  (0.51) |
| Budget NEU | 40.73  (2.6) | 38.98 (3.14) | 38.44 (2.26) | 34.97 (2.93) | 41.4 (2.99) | 39.85  (3.8) | 36.45 (3.05) | 29.81 (1.91) |

*Supplementary Table 5(a).* Total use of resources (tons) in the CE economy/Total use of resources (tons) in the scenario with the increasing wage gap between EU and NEU

|  | CE EU only | CE EU only    Slow budget updating | CE EU only    Fast budget  updating | Global CE | Global CE  Slow budget  updating | Global CE  Fast budget updating |
| --- | --- | --- | --- | --- | --- | --- |
| Aluminum | 1.08*** | 1.10*** | 1.36*** | 3.13*** | 3.77*** | 4.71*** |
| Copper | 1.09*** | 0.47*** | 0.60*** | 1.70*** | 0.65*** | 0.84*** |
| Iron | 1.14*** | 1.18*** | 1.47*** | 4.56*** | 5.15*** | 6.35*** |
| Lead, zinc, tin | 1.08*** | 0.76*** | 0.72*** | 1.61*** | 1.17*** | 1.04** |
| Non-ferrous  metals | 1.04*** | 1.02 | 1.19*** | 4.20*** | 4.07*** | 4.34*** |
| Forest | 1.06*** | 1.08*** | 1.32*** | 1.36*** | 1.62*** | 1.99*** |
| Coal | 1.03** | 1.05*** | 1.29*** | 1.02 | 1.21*** | 1.49*** |
| Petroleum | 1.04** | 1.08*** | 1.35*** | 1.03 | 1.25*** | 1.57*** |
| Gas | 1.04*** | 1.09*** | 1.34*** | 1.06*** | 1.29*** | 1.60*** |
| Electricity EU | 1.00 | 0.81*** | 0.98** | 0.99** | 0.92*** | 1.12*** |
| Electricity NEU | 1.04*** | 1.12*** | 1.40*** | 1.06*** | 1.33*** | 1.67*** |
| Global spending | 1.05** | 1.17*** | 1.49*** | 1.06** | 1.39*** | 1.77*** |
| Budget EU | 1.01* | 0.81*** | 0.99* | 1.01 | 0.93*** | 1.14*** |
| Budget NEU | 1.05** | 1.25*** | 1.61*** | 1.07** | 1.49*** | 1.93*** |

*Supplementary Table 5(b).* Resource use in different scenarios **(**Million tons/ Million Tera Joules/ Billion Euros); in the scenario with the increasing wage gap between EU and NEU

|  | No CE | CE EU only | CE EU only;  Slow budget updating | CE EU only;  Fast budget  updating | Global CE | Global CE;  Slow budget  updating | Global CE;  Fast  budget updating |
| --- | --- | --- | --- | --- | --- | --- | --- |
| Aluminum | 50.05 (4.17) | 46.44 (4.31) | 45.44 (4.96) | 36.75 (3.27) | 15.97 (1.71) | 13.28 (1.61) | 10.63 (1.11) |
| Copper | 20.9  (1.64) | 19.09 (1.93) | 44.33 (5.51) | 34.83 (3.48) | 12.26  (1.3) | 31.95 (4.08) | 24.77 (2.78) |
| Iron | 983.3 (82.69) | 862.96 (81.29) | 831.65 (91.03) | 669.55 (59.3) | 215.63 (21.63) | 190.95 (23.26) | 154.85 (17.36) |
| Lead, zinc, tin | 13.04 (1.03) | 12.06 (1.06) | 17.15 (2.13) | 18.01  (1.8) | 8.11  (0.83) | 11.17  (1.5) | 12.48 (1.44) |
| Non-ferrous  metals | 21.12 (1.77) | 20.33 (1.87) | 20.68 (2.31) | 17.68 (1.61) | 5.03  (0.53) | 5.19  (0.64) | 4.87  (0.53) |
| Forest | 1803.19 (144.12) | 1702.96 (151.22) | 1668.02 (177.7) | 1367.51 (119.01) | 1327.46 (136.05) | 1114.51 (120.11) | 904.12 (89.52) |
| Coal | 8693.07 (697.31) | 8423 (741.24) | 8277.65 (890.63) | 6719.5 (594.17) | 8486.23 (871.65) | 7200.51 (777.28) | 5818.06 (582.95) |
| Petroleum | 8569.28 (726.66) | 8268.26 (770.31) | 7912.89 (887.45) | 6355.4 (586.02) | 8333.85 (901.87) | 6846.25 (774.09) | 5464.58 (569.74) |
| Gas | 5196.75 (430.51) | 4992.62 (455.49) | 4787.49 (525.81) | 3890.21 (350.41) | 4884.92 (517.93) | 4028.63 (445.56) | 3249.93 (330.99) |
| Electricity EU | 10.26 (0.46) | 10.23 (0.51) | 12.68 (1.18) | 10.45 (0.81) | 10.36 (0.66) | 11.11 (1.04) | 9.14  (0.81) |
| Electricity NEU | 57.48 (5.05) | 55.22 (5.34) | 51.21 (5.76) | 41.2  (3.8) | 54.35 (6.08) | 43.23 (4.91) | 34.5  (3.59) |
| Global spending | 92.05 (10.11) | 87.81 (10.4) | 78.72 (9.56) | 61.59 (5.97) | 86.73 (11.33) | 66.45 (8.04) | 51.96 (5.33) |
| Budget EU | 11.66 (0.55) | 11.53  (0.6) | 14.46 (1.36) | 11.75 (0.88) | 11.49 (0.75) | 12.55 (1.16) | 10.24 (0.82) |
| Budget NEU | 80.39 (9.56) | 76.28  (9.8) | 64.26  (8.2) | 49.84 (5.09) | 75.24 (10.58) | 53.9  (6.88) | 41.72 (4.51) |

*Supplementary Table 6(a).* Total use of resources (tons) in the CE economy/Total use of resources (tons) in the scenario characterized by closing the wage gap between EU and NEU

|  | CE EU only | CE EU only    Slow budget updating | CE EU only    Fast budget  updating | Global CE | Global CE  Slow budget  Updating | Global CE  Fast budget updating |
| --- | --- | --- | --- | --- | --- | --- |
| Aluminum | 1.07*** | 0.95*** | 0.99 | 3.05*** | 3.04*** | 3.09*** |
| Copper | 1.03** | 0.53*** | 0.55*** | 1.47*** | 0.70*** | 0.71*** |
| Iron | 1.14*** | 1.03** | 1.09*** | 4.07*** | 3.57*** | 3.65*** |
| Lead, zinc, tin | 1.07*** | 0.82*** | 0.64*** | 1.50*** | 1.15*** | 0.81*** |
| Non-ferrous  metals | 1.01 | 0.90*** | 0.89*** | 3.93*** | 3.56*** | 3.04*** |
| Forest | 1.04*** | 0.94*** | 0.97** | 1.31*** | 1.30*** | 1.32*** |
| Coal | 1.01 | 0.94*** | 0.97** | 0.99 | 0.99 | 1.02 |
| Petroleum | 1.01 | 0.95*** | 0.99 | 0.99 | 1.01 | 1.04** |
| Gas | 1.02 | 0.95*** | 0.99 | 1.02 | 1.04** | 1.06*** |
| Electricity EU | 1.00 | 0.96*** | 1.02 | 0.97** | 1.03** | 1.08*** |
| Electricity NEU | 1.01 | 0.94*** | 0.99 | 1.02 | 1.03** | 1.06*** |
| Global spending | 1.01 | 0.98** | 1.04** | 1.00 | 1.05*** | 1.10*** |
| Budget EU | 1.00 | 0.97** | 1.04** | 0.99 | 1.05*** | 1.11*** |
| Budget NEU | 1.02 | 0.98* | 1.03** | 1.01 | 1.06*** | 1.09*** |

*Supplementary Table 6(b).* Resource use in different scenarios **(**Million tons/ Million Tera Joules/ Billion Euros) in the scenario characterized by closing the wage gap between EU and NEU

|  | No CE | CE EU only | CE EU only;  Slow budget updating | CE EU only;  Fast budget  updating | Global CE | Global CE;  Slow budget  updating | Global CE;  Fast  budget updating |
| --- | --- | --- | --- | --- | --- | --- | --- |
| Aluminum | 37.09 (3.03) | 34.82 (3.13) | 39.11 (2.94) | 37.42 (3.41) | 12.15 (1.19) | 12.19 (1.28) | 12.02 (1.35) |
| Copper | 16.28 (1.32) | 15.85 (1.61) | 30.85 (2.31) | 29.49 (2.71) | 11.05  (1.2) | 23.39  (2.4) | 22.86 (2.55) |
| Iron | 724.32 (58.11) | 634.83 (55.77) | 702.19 (51.67) | 667.39 (59.4) | 178.07 (17.58) | 203.15 (21.37) | 198.53 (23.13) |
| Lead | 10.26 (0.85) | 9.62  (0.88) | 12.55 (0.98) | 16.14 (1.52) | 6.83  (0.68) | 8.94  (0.99) | 12.66 (1.47) |
| Non-ferrous  metals | 15.62 (1.27) | 15.4  (1.37) | 17.32  (1.3) | 17.47 (1.59) | 3.97  (0.38) | 4.39  (0.46) | 5.14  (0.58) |
| Forest | 1364.5 (108.64) | 1306.15 (115.16) | 1459.33 (107.91) | 1412.71 (127.06) | 1044.49 (97.86) | 1047.55 (105.71) | 1030.99 (111.19) |
| Coal | 6735.82 (550.72) | 6683.02 (598.03) | 7190.89 (535.35) | 6910.69 (649.87) | 6833.06 (648.94) | 6775.58 (685.33) | 6635.85 (727.82) |
| Petroleum | 6398.72 (546.17) | 6332.9 (592.59) | 6733.31 (523.97) | 6437.42 (608) | 6485.34 (643.24) | 6327.25 (670.95) | 6167.16 (695.57) |
| Gas | 3899.46 (323.42) | 3834.07 (349.73) | 4102.67 (311.56) | 3950.8 (363.88) | 3813.79 (368.33) | 3739.12 (386.5) | 3664.94 (403.14) |
| Electricity EU | 12.79 (1.12) | 12.81 (1.18) | 13.36 (1.03) | 12.54 (1.15) | 13.12 (1.32) | 12.4  (1.29) | 11.84  (1.3) |
| Electricity NEU | 39.33  (3.2) | 38.78  (3.5) | 41.72 (3.13) | 39.9  (3.64) | 38.7  (3.68) | 38.21 (3.91) | 37.23 (4.06) |
| Global spending | 58.82 (5.13) | 58.18 (5.37) | 60.12 (4.65) | 56.73 (5.23) | 58.68  (5.8) | 55.79 (5.71) | 53.66 (5.68) |
| Budget EU | 16.36 (1.59) | 16.34 (1.61) | 16.93  (1.4) | 15.7  (1.53) | 16.57  (1.8) | 15.54 (1.68) | 14.68 (1.62) |
| Budget NEU | 42.46 (3.54) | 41.84 (3.76) | 43.19 (3.25) | 41.03  (3.7) | 42.11  (4) | 40.25 (4.03`) | 38.98 (4.06) |
